# Specialized Biopsychosocial Care in Inpatient Somatic Medicine Units—A Pilot Study

**DOI:** 10.3389/fpubh.2022.844874

**Published:** 2022-04-12

**Authors:** Paul Köbler, Eva K. Krauss-Köstler, Barbara Stein, Joachim H. Ficker, Martin Wilhelm, Alexander Dechêne, Christiane Waller

**Affiliations:** ^1^Department of Psychosomatic Medicine and Psychotherapy, Paracelsus Medical Private University, Nuremberg General Hospital, Nuremberg, Germany; ^2^Department of Internal Medicine 3, Respiratory Medicine, Paracelsus Medical Private University, Nuremberg General Hospital, Nuremberg, Germany; ^3^Department of Internal Medicine 5, Oncology/Hematology, Paracelsus Medical Private University, Nuremberg General Hospital, Nuremberg, Germany; ^4^Department of Internal Medicine 6, Gastroenterology, Paracelsus Medical Private University, Nuremberg General Hospital, Nuremberg, Germany

**Keywords:** integrated care, biopsychosocial approach, psychosomatics, internal medicine, chronic disease, Psychiatric Medicine Units, psychotherapy

## Abstract

**Introduction:**

Specialized biopsychosocial care concepts are necessary to overcome the dualism between physical and psychosocial treatment in acute care hospitals. For patients with complex and chronic comorbid physical and mental health problems, neither standardized psychiatric/psychosomatic nor somatic care units alone are appropriate to their needs. The “***N***uremberg ***I***ntegrated ***P***sychosomatic ***A***cute Unit” (NIPA) has been developed to integrate treatment of both, psychosocial and physical impairments, in an acute somatic care setting.

**Method:**

NIPA has been established in inpatient internal medical wards for respiratory medicine, oncology and gastroenterology. One to two patients per ward are regularly enrolled in the NIPA treatment while remaining in the same inpatient bed after completion of the somatic care. In a naturalistic study design, we evaluated treatment effects by assessment of symptom load at admission and at discharge using the Patient Health Questionnaire (PHQ) and the Generalized Anxiety Disorder Scale-7 (GAD-7). Furthermore, we assessed the severity of morbidity using diagnosis data during treatment. At discharge, we measured satisfaction with treatment through the Patient Satisfaction Questionnaire (ZUF-8).

**Results:**

Data from 41 NIPA patients were analyzed (18–87 years, 76% female). Seventy-eight percent suffered from at least moderate depression and 49% from anxiety disorders. Other diagnoses were somatoform pain disorder, somatoform autonomic dysfunction, eating disorder and posttraumatic stress disorder. Hypertension, chronic lung diseases and musculoskeletal disorders as well as chronic oncological and cardiac diseases were the most common somatic comorbidities. Treatment resulted in a significant reduction of depressive mood (admission: *M* = 10.9, *SD* = 6.1, discharge: *M* = 7.6, *SD* = 5.3, *d* = 0.58, *p* = *0.001*), anxiety (admission: *M* = 10.6, *SD* = 4.9, discharge: *M* = 7.3, *SD* = 4.1, *d* = 0.65, *p*< *0.001*) and stress (admission: *M* = 6.0, *SD* = 3.6, discharge: *M* = 4.1, *SD* = 2.5, *d* = 0.70, *p*< *0.001*). Somatic symptom burden was reduced by NIPA treatment (admission: *M* = 10.9, *SD* = 5.8, discharge: *M* = 9.6, *SD* = 5.5, *d* = 0.30), albeit not statistically significant (*p* = *0.073*) ZUF-8 revealed that 89% reported large or full satisfaction and 11% partial dissatisfaction with treatment.

**Discussion:**

NIPA acute care is bridging the gap for patients in need of psychosocial treatment with complex somatic comorbidity. Further long-term evaluation will show whether psychosocial NIPA care is able to reduce the course of physical illness and hospital costs by preventing hospitalization and short-term inpatient re-admissions.

## Introduction

Worldwide, there are proven and established treatment structures regarding somatic issues on one hand and psychosomatic-psychiatric care on the other. However, a high prevalence of mental comorbidities is present in patients with chronic and complex somatic diseases affecting daily clinical practice. Studies show regularly an elevated somatic comorbidity in people with psychiatric and psychosomatic diagnoses and higher rates of manifest mental illnesses in patients with physical disorders (1, 2).

A meta-analysis estimated the prevalence of clinically relevant depression in patients suffering from chronic obstructive pulmonary disease (COPD) to be 40% and the prevalence of anxiety and panic disorders to be 37% (3, 4). The prevalence of depressive disorders is estimated to be about 20% in patients with heart failure and coronary artery disease, which is 2 to 4 times higher than in the general population (5–7). Additionally, other diagnoses e.g. posttraumatic stress or bodily distress disorder are regularly seen in chronic physical diseases (8, 9). Other psychosomatic syndromes can also significantly increase the symptom severity and suffering in somatic diseases. Patients with, e.g. eating disorders, show a high prevalence of typical gastrointestinal symptoms such as constipation or diarrhea up to ileus-like symptoms which are difficult to treat without knowledge of the underlying psychosocial problems (10).

These observations lead to a constant adaptation of the health system to define and characterize psychosomatic complexity, which is represented by an update of the international classification of diseases. With new diagnostic entities (DSM V: Somatic Symptom Disorder, ICD 11: Bodily Distress Disorder), the high degree of overlap and mutual interaction of psychological and physical symptoms, especially in chronic conditions such as cardiovascular, lung or cancer diseases, is taken into account (11–14). Attention to these clinical phenomena is highly needed, considering elevated mortality rates, functional impairment and societal costs due to lost workdays and greater utilization of health care associated with psychiatric and psychosomatic comorbidity in somatic patients (15–17). In addition, prolonged hospitalization for mental disorders has been demonstrated for some constellations (18).

In everyday clinical practice, these implications can be observed frequently and in a wide variety of manifestations, e.g. intense anxiety reduces self-management skills in dealing with the somatic disease. This can be illustrated by COPD patients fearing physical exertion and therefore tend to be less willing to exercise or withdraw from active daily life due to fears of social stigmatization (19).

Depressive symptoms such as anhedonia or lack of interest, social withdrawal and sleep disturbances are associated with a significantly reduced quality of life of patients and result in an impaired ability to actively cope with the disease. Panic attacks occurring in the context of anxiety disorders with somatic comorbidity often lead to repetitive emergency room admission and inpatient treatment, increasing the risk of worsening the underlying chronic disease. Recent research has shown that depressive symptoms are associated with a higher risk of COPD exacerbation and poorer prognosis and therefore highlights the need for psychotherapeutic (co-) treatment (19–21). Strong associations between psychological distress and treatment results, disease progression, and quality of life have also been shown for coronary artery disease (7, 22). Both depressive and anxiety disorders are associated with an increased risk of cardiovascular morbidity and mortality [e.g. (23)]. Accordingly, the related statement of the German Society of Cardiology explicitly recommends psychotherapeutic interventions to address these associations (24). Furthermore, there are patients with somatization on internal wards, who show twice as many utilizations and medical care costs than non-somatizing patients (25).

Psychiatric and psychosomatic comorbidities are usually not diagnosed in the common somatic setting, so that these disorders often become chronic (15, 26–28) and result in repetitive and mismatched inpatient admission and treatment with increasing overall financial costs for the health system (29, 30). These patients have rather the problem of refused admission to medical wards (when mental comorbidity is striking) and to psychosomatic or psychiatric wards (when somatic symptoms are too severe) and sometimes even suffer from premature discharge because of difficulties in treatment within the framework of traditional care structures (31). When psychosomatic or psychiatric comorbidities interfere with care, treatment staff can quickly become overwhelmed, which in turn leads to shortages of care for this patient population (32).

According to Huyse and Stiefel, complex medically ill patients benefit from “complexity models, such as the biopsychosocial model—which focuses more on interactions such as compliance, the quality of the patient–doctor relationship, or interdependence between psychological stressors and physical disorders, rather than on separate disease identities [to] enrich the quality of service delivery to these patient groups” [(33), p. 257].

### Biopsychosocial Inpatient Treatment Models

Following a short overview of the existing integrated care models and their development, the NIPA concept is presented as an innovative psychosomatic treatment unit and sorted into the previous models in terms of its ability to close the gap in biopsychosocial care for the chronically and complex medically ill. Initial clinical data evaluating treatment effect complete the report.

There have been efforts around the world in recent decades to enhance integrated care models, which are defined as coordinated care between general and mental health as well as social service disciplines. There is a wide range of models that differ in terms of the manner of collaboration between these care providers (34). Inpatient integrated treatment is particularly necessary when outpatient care is insufficient due to the severity, complexity or acute nature of the complaints and when multimodal treatment is needed (1). The first milestone in this integrated perspective was the development and expansion of consultation and liaison services (C-L) of psychiatry in general hospitals, which was started in the 1950s/60s in the USA and established in the following decades in Europe, Asia and Oceania as well as in South America (35). There are also forms of integration that provide for physical health liaison within mental health settings (36). Kathol et al. present a four-level categorization of integrated treatment models, which are termed Medical Psychiatry Units or Psychiatric Medicine Units, and are referenced by treatment programs worldwide (37). Type I and Type II follow the traditional approach and are most likely to be represented by psychiatric wards with somatic C-L support (Type I) or general hospital wards with psychiatric C-L support (Type II) (38, 39). Type III and IV would care for patients with moderate to severe psychiatric and somatic symptom severity in a stronger organizational integration whereas Type IV units “can diagnose and treat the same medical problems as general-medicine wards, regardless of the severity, together with any psychiatric condition generally handled in an acute-care psychiatric ward” [(31), p. 355]. Since the type definitions overlap and are sometimes not distinctive enough (40), the summarizing term Complexity Intervention Unit, which was introduced by Kathol et al. in 2009 (41) seems more appropriate for all these units providing more integrated biopsychosocial care. All over the world these kinds of clinical organizations were developed, differing in administration (psychiatry or general medicine), location (e.g., public, academic or private hospital) and specification (e.g., specific chronic conditions or disorders) (42). The vast majority of these units are more or less large bed units that allow for multimodal treatment of mental as well as somatic comorbidities (e.g., by lockable rooms on the one hand and oxygen supplies on the other). Illustrative examples can be found, among many others, in Alberque et al. (31) (USA), Wullschleger et al. (43) (Switzerland), Buckley et al. (44) (Ireland), Leue et al. (45) (Netherlands), Gertler et al. (46) (Australia) or Nomura et al. (47) (Japan). The most numerous efforts in this regard are directed toward the integrated care of severe mental illnesses especially schizophrenia, bipolar disorder and major depressive disorder (2, 48).

In Germany, a particular development of integrated medicine influenced by psychodynamic theory resulted in the establishment of the independent medical specialty of psychosomatic medicine with its distinct care units (35, 49). Modern psychosomatic therapy has since integrated a variety of method- and disorder-specific techniques drawn from a number of approved therapeutic disciplines (50). Comparable developments of independent psychosomatic specialties can be found in a number of countries, such as the other German-speaking states of Switzerland and Austria, but also, for example, in Japan and the Baltic states (51–54). The population treated in psychosomatic care is slightly different to Medical Psychiatry Units e.g., in the United States. The most prevalent diseases which are treated in psychosomatic wards in Germany are affective and anxiety disorders as well as somatoform disorders, eating disorders and trauma- and stress-related disorders in a specialized group setting with a comprehensive treatment plan (35, 50). Meta-analyses showed moderate to strong treatment effects of inpatient psychosomatic treatment in terms of symptom severity, well-being, and overall functioning (55, 56). However, complex and chronic somatically ill patients often do not fit to these standard psychosomatic wards due to the severity of their physical comorbidities and their need of continuous and specialized somatic co-care.

**Figure 1:**
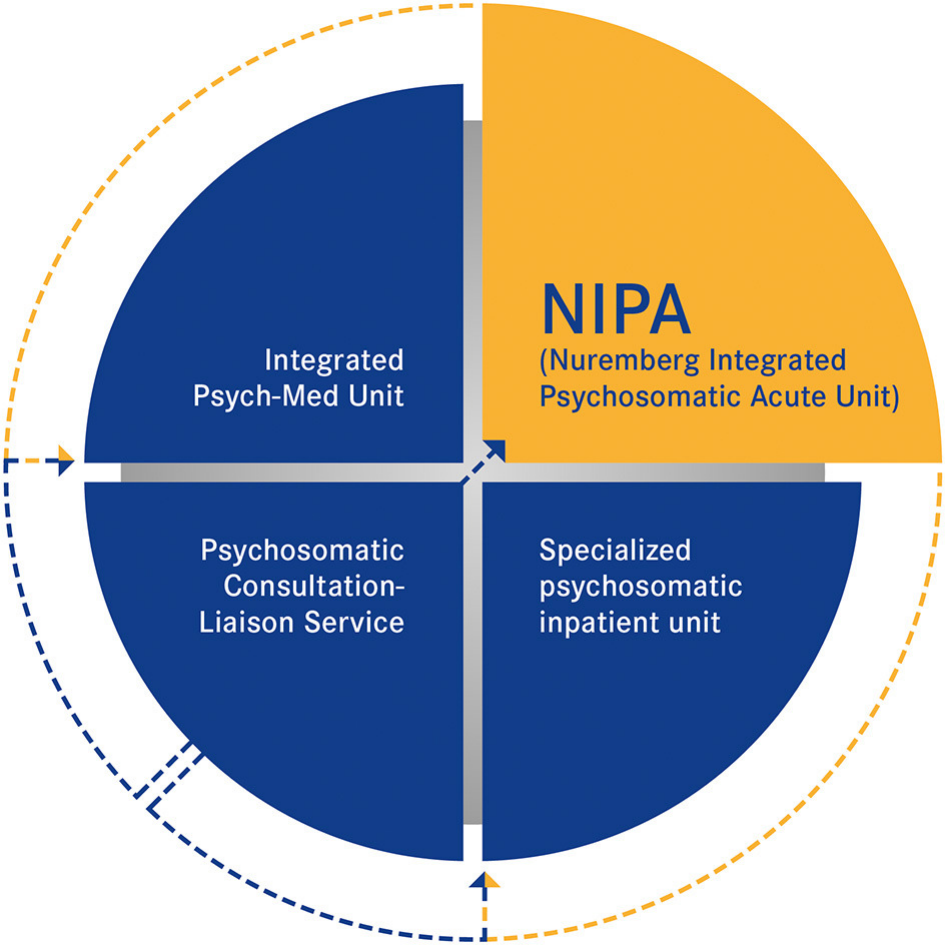
Illustration of the treatment gap in the biopsychosocial care of psychosomatic patients in Germany. NIPA represents an important component for rounding off the infrastructure in healthcare for the complex medically ill. Referral is made by the CL service from the internal medicine wards of general hospital. NIPA, in turn, can be a “door opener” to further psychosomatic treatment programs if needed.

In line with the presented developments in integrated care, models have been established to realize an inpatient biopsychosocial based approach which exceeds the general treatment options of typical specialized psychosomatic inpatient units (Figure 1, bottom right). These units do not provide adequate internal medicine care competence and equipment appropriate to complex medically ill patients. In Nuremberg General Hospital, like in many others around Germany, a psychosomatic C-L service is integrated throughout the hospital and enables comprehensive psychosomatic co-care of complex and chronically diseased patients by specialized staff assigned to the departments (35, 57) (Figure 1, bottom left). For patients with higher biopsychosocial treatment needs, this therapy model is not always sufficient. Building on this, a specialized inpatient Psychiatric Medicine Unit for biopsychosocial care of the comorbid somatic and mentally ill patients has been successfully established (57) (Figure 1, top left). In this unit, patients are treated who require regular somatic co-care but who are resilient enough to fit effectively into a psychotherapeutic treatment program lasting 6 to 8 weeks consisting of group-based interventions.

Finally, there remains a gap in the care of chronically ill and often complex patients who need psychotherapeutic treatment, because they are often too impaired to participate in this kind of intensive treatment plan, especially in a group setting. For these patients, all kinds of established psychosomatic inpatient, but also outpatient care is often inaccessible, in particular due to limited mobility. Furthermore, these patients often have no idea how to benefit from biopsychosocial support because of lack of psychotherapeutic experience, which often results in reduced motivation for therapy. Additionally, the experience of psychotherapists in working with physically ill patients is generally low.

Another major challenge in accessing these patients are the long waiting times for psychosomatic treatment, often lasting several weeks to months. For complexly ill, mentally and somatically impaired patients, however, longer waiting times often mean a prolongation of hospitalization in somatic wards, which worsens the course of the disease, as explained above. A more rapid treatment perspective is therefore urgently needed. The Nuremberg Integrated Psychosomatic Acute Unit (NIPA) is trying to improve treatment options regarding all these points and therefore, closes the gap in biopsychosocial care in Germany (Figure 1, top right).

As for evaluation, we would like to explore whether NIPA treatment is resulting in a reduction of symptom burden and is well accepted by the patient group. Thus we hypothesize that anxiety and depressiveness and stress can be significantly reduced and satisfaction with the treatment is high.

## Materials and Methods

### Clinical Setting

Since 2018, NIPA has been established in inpatient wards of the departments for respiratory medicine, oncology and gastroenterology. One to two patients per ward are regularly included in the NIPA treatment while remaining in the same inpatient bed after somatic stabilization. This approach enables the acute admission of patients to psychosocial mental health care and increases the patient's compliance and motivation since the treatment setting continues to include specialized somatic treatment. For example, hospitalized COPD patients with frequent recurrent inpatient treatments (19) benefit more from this approach.

The initiative for psychosocial co-treatment is taken by the internal medicinal referral to a psychosomatic consultation—liaison service for assessment, partially in the course of a proactive consultation model to improve case detection (41). In case of relevant psychosomatic comorbidity, a psychosomatic-psychotherapeutic treatment in NIPA is planned. After improvement of somatic complaints, psychosomatic conditions are the leading cause for hospitalization and patients are included in NIPA treatment.

An individual treatment plan is drawn up in line with therapy goals, which are achieved by low-threshold, psychoeducational and practice-based interventions. The aim of this biopsychosocial approach is 1. to provide acute and low-threshold psychosocial support and 2. to serve as a “door-opener” for further specialized psychosocial mental health treatment in the outpatient or day-care sector. This is realized by extending the patients' disease model by focusing on psychosomatic and psychosocial understanding of disease processes, supporting stabilization and resource activation. The important psychoeducational content is based especially on clarifying psychosomatic relationships between anxiety, tension and stress with bodily signals such as dyspnea and pain, as well as showing the effectiveness of relaxation on the organism, combined with experiential exercises (e.g., relaxation and imagination techniques). In NIPA, resource activation is primarily focused on those areas of life that are important to the patients and addresses how participation can still be achieved despite limitations—perhaps with the help of additional resources or with a slightly different intensity than before. Another important component is the optimization of drug therapy, e.g. with regard to improving sleep or energy. Physical therapy units aim at enhancing mobilization and movement. The NIPA treatment modules are designed to motivate chronically ill patients without overwhelming them. The multi-professional psychosocial team consists of medical, psychological, art therapeutic, body therapeutic, physiotherapeutic and nursing professional staff. Another important treatment module is the co-management by social services, that focuses on improvement of domestic care as well as on helping with the integration to diverse community offers (e.g., outpatient social psychiatry services) (Table 1).

**Table 1:** NIPA treatment modules.

**Intervention**	**Details**
Psychosomatic medical round	
Psychotherapy	e.g., Psychoeducation, development of psychosomatic disease model, motivation, psychosocial interventions
Psychosomatic nursing	e.g., Therapeutic diary, collecting positive experiences, training of adaptive sleeping or nutrition routines
Physical therapy	e.g., Mobilization, respiratory therapy
Relaxation techniques	e.g., PMR, imagination
Art- or body therapy	Therapy with perception and expression of feelings, thoughts and actions through movement and body experience or creative work through visual art media.
Social service	e.g., Support in applying for assistance regarding domestic care, integration to diverse outpatient community offers

The psychosomatic therapy program is continuously accompanied by daily medical and nursing care of the somatic ward. By virtue of close, interdisciplinary exchange, a quick response to any changes in the general somatic condition can be guaranteed through specialized somatic care.

### Psychometric Instruments

In a naturalistic study design, we assessed the severity of morbidity using diagnosis data during treatment. The severity of mental health problems at admission and at discharge was measured using the Patient Health Questionnaire [PHQ (58, 59)] and Generalized Anxiety Disorder Scale-7 [GAD-7 (60)]. Furthermore, a general assessment of treatment, the relevance to patient needs and satisfaction with treatment were evaluated using the Patient Satisfaction Questionnaire [ZUF-8 (61)].

### Statistical Analyses

A Wilcoxon signed-rank test was calculated to compare the severity of symptoms at admission and discharge. Data are presented in mean (M) and standard deviation (SD) with *p* < 0.05 considered statistically significant. Statistical power (d) was additionally calculated. In a second step, we calculated the effective symptom changes of the individual patients by a description of frequencies in the sample. Both PHQ (PHQ9/PHQ15) as well as GAD-7 enabled this by categorization of symptom severity (severe, moderate, mild, normal symptom manifestation). We described patients with improvement over two categories as having major improvement, and patients with improvement over one category as having moderate improvement. Satisfaction of treatment (ZUF-8) was analyzed by a description of frequencies in the sample.

## Results

Until May 2021 we treated 41 patients in NIPA for an average of 15.7 days (*SD* = 5.3, min = 2, max = 29). The mean age of the patients was 59.9 years (18-87 years). Thirty-one patients were female (76%) and 10 male (24%). The sample showed a high rate of various mental and somatic diagnoses (Table 2). Besides the main psychosomatic diagnosis, the median of mental comorbidities was 1 (min = 0; max = 4), the median of somatic comorbidities was 9 (min = 2, max = 25). We included 38 patients in the analysis of the treatment outcome (admission vs. discharge) due to missing data regarding the symptom evaluation at discharge from three patients (two because of premature dropout from treatment, one because of necessary transfer to intensive care unit due to somatic symptom worsening).

**Table 2:** Diagnoses in NIPA treatment.

***n* = 41**	**n**	**%**
**Mental disorders**		
Depression		
*Depressive episodes, recurrent depressive disorder (mild–severe)*	32	78,0
Anxiety		
*Phobias, Panic Disorder*	20	48,8
Somatoform disorders		
*Somatization Disorder, Somatoform autonomic dysfunction, Persistent somatoform pain disorder*	14	34,1
Eating disorders		
*Anorexia nervosa*	2	4,9
**Somatic comorbidities**		
Limitations in mobility/dependence on medical devices	23	56,1
Hypertension	23	56,1
Chronic lung disease		
*COPD, Emphysema, Asthma, Chronic respiratory failure, Bronchiectasis, etc*.	20	48,8
Gastrointestinal diseases		
*Chronic or acute gastritis, Diverticulitis, Constipation, Nausea, etc*.	20	48,8
Musculoskeletal disorders		
*Osteoporosis, Fibromyalgia, Dorsalgia, etc*.	16	39,0
Urogenital diseases		
*Hyperplasia of prostate, Retention of urine, Anuria, Vesicointestinal fistula* etc.	10	24,4
Cachexia	10	24,4
Oncological diseases		
*Malignant neoplasm, secondary neoplasm*	9	22,0
Cardiac diseases		
*Heart failure, Ischemic heart diseases, Persistent atrial fibrillation, etc*.	9	22,0
Thyroid diseases		
*Hypothyroidism, thyroid nodule*	9	22,0
Diabetes	8	19,5
Vascular diseases		
*Atherosclerosis*	4	9,8
Nephrological diseases		
*Chronic kidney disease, unspecified kidney failure, acute renal failure*	3	7,3

**Figure 2:**
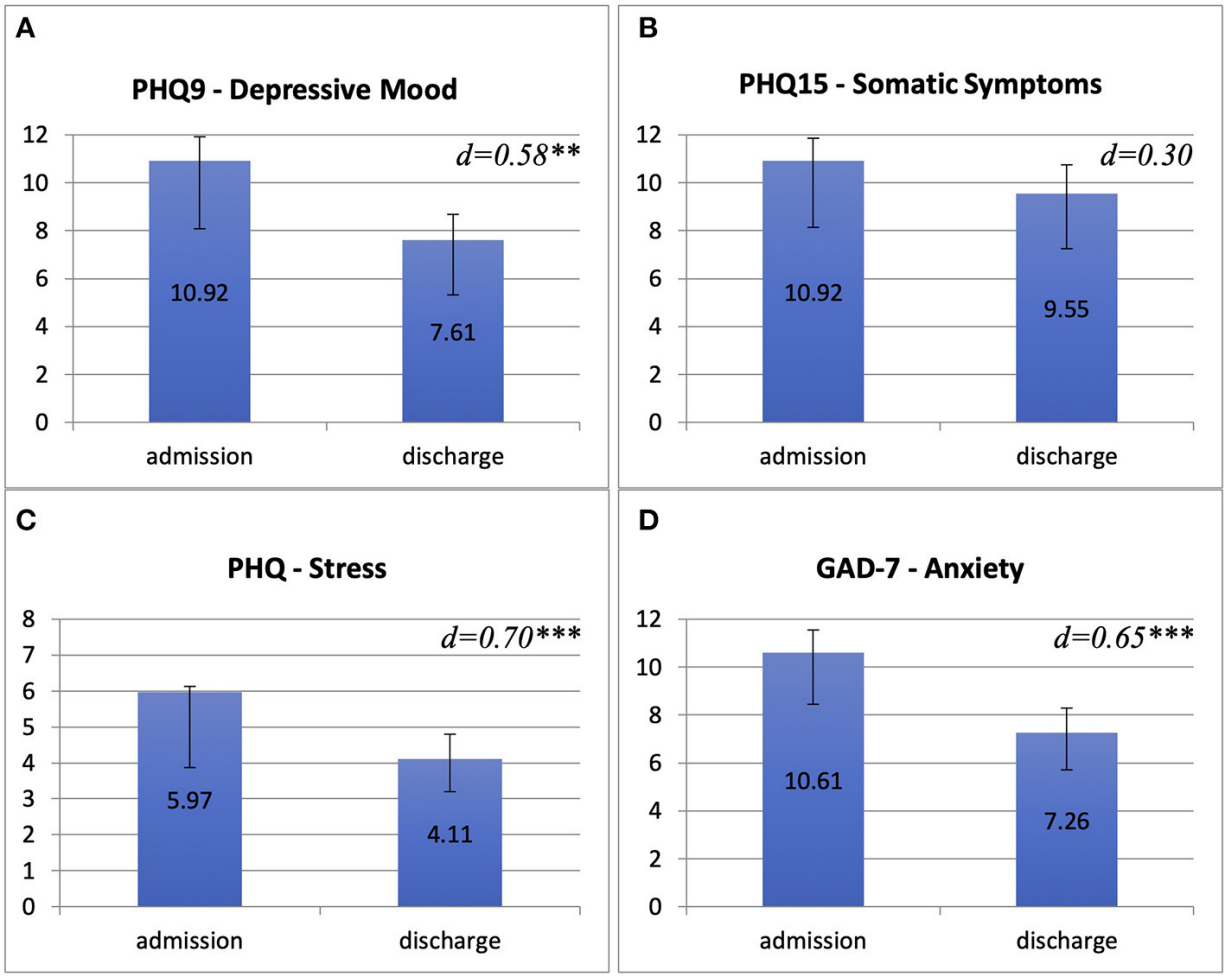
NIPA treatment outcomes compared between admission and discharge on the four symptom scales assessed. We compared means (Y-axis) of Patient Health Questionnaire for depressive mood [PHQ9, **(A)**], Patient Health Questionnaire for somatic symptoms [PHQ15, **(B)**], PHQ-stress-scale **(C)** and Generalized Anxiety Disorder Scale-7 [GAD-7, **(D)**].

Treatment (*n* = 38) resulted in a significant reduction of depressive mood (admission: *M* = 10.9, *SD* = 6.1, discharge: *M* = 7.6, *SD* = 5.3, *d* = 0.58, *p* = *0.001, PHQ9*), anxiety (admission: *M* = 10.6, *SD* = 4.9, discharge: *M* = 7.3, *SD* = 4.1, *d* = 0.65, *p*< *0.001, GAD-7*) and stress (admission: *M* = 6.0, *SD* = 3.6, discharge: *M* = 4.1, *SD* = 2.5, *d* = 0.70, *p*< *0.001, PHQ*). Changes in somatic symptom burden were not significant (admission: *M* = 10.9, *SD* = 5.8, discharge: *M* = 9.6, *SD* = 5.5, *d* = 0.30, *p* = *0.073, PHQ15*) (Figure 2).

In terms of categorial symptom changes regarding the whole sample, in depressive symptoms (PHQ9) seven patients (18.4%) showed major and 11 (28.9%) showed moderate improvement whereas three patients (7.9%) showed minor worsening and one patient (2.6%) showed major worsening. Sixteen patients (42.1%) reported no categorial changes in depressive symptoms. Eight patients (21.1%) showed major and 14 patients (36.8%) showed moderate improvement in anxiety symptom burden (GAD-7), whereas two patients (5.3%) reported minor worsening, one patient (2.6%) reported major worsening and 13 patients (34.2%) were unchanged. In somatic symptoms (PHQ15) we found moderate improvement in 16 patients (42%), minor worsening in six patients (15.8%), major worsening in two patients (5.3%) and no categorial change in 14 patients (36.8%). In total, four patients (10.5 %) reported no improvement in any of the outcome measures. Out of these, two showed a symptom increase on all scales.

**Figure 3:**
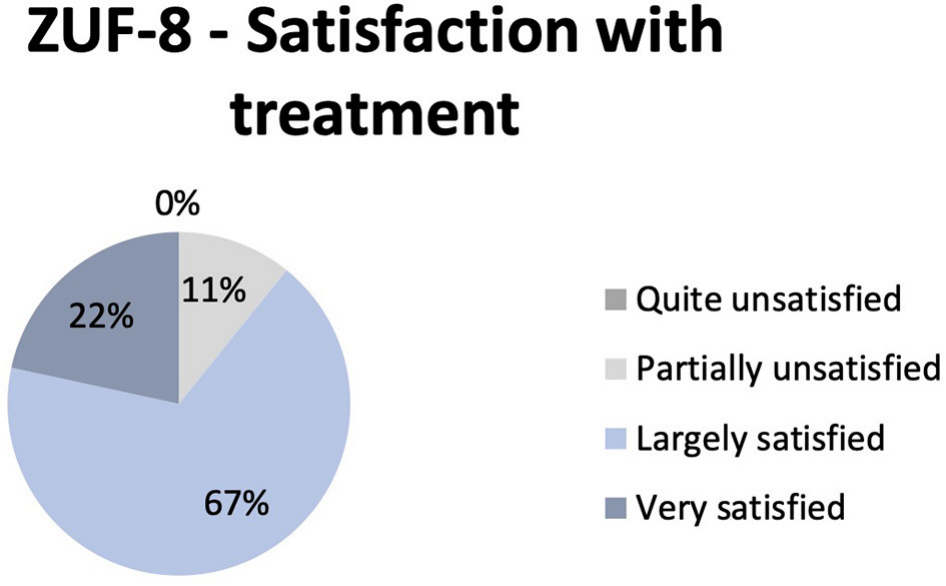
Reported satisfaction with NIPA treatment as measured by Patient Satisfaction Questionnaire (ZUF-8). Diagram shows the frequency of the different levels of satisfaction.

Eighty-nine percent of patients reported large or full satisfaction with treatment (ZUF-8) (somewhat or very helpful in dealing with their problems; therapy meeting most or almost all of their needs, respectively). No patient rated the therapy unsatisfactory, although 11% of patients reported partial dissatisfaction across all diagnosis groups. Key items regarding this dissatisfaction were 1. because they expected greater extent of support regarding their somatic issues and 2. wished for more assistance in dealing with their resulting problems appropriately (Figure 3).

## Discussion

Our clinical experience with the NIPA concept to date shows advantages for both patients and treatment providers. Firstly, it offers biopsychosocial stressed, underserved patient groups the chance of specialized and individualized psychosomatic-psychotherapeutic treatment. Secondly, it expands the intervention repertoire of psychosomatic clinics in the urgently needed expansion of treatment capacities for somatically ill patients. In terms of categorization according to Kathol et al. (37), the NIPA concept can be classified a type III Psychiatric Medicine Unit, as it is a highly specialized integrated treatment context, but does not treat medical patients with high acuity. Thus, it can be well-described by the more modern term Complexity Intervention Unit, which is characterized, in particular, by administration through an alliance of general and psychosomatic care, location in general hospital with physical and mental health safety features and capabilities to treat patients with high health complexity (41). Most of these Complexity Intervention Units worldwide focus on integrated treatment of severe mental illnesses e.g., to provide acute medical care for people with serious psychotic symptoms. NIPA, by comparison, follows rather the psychosomatic treatment approach, which has its unique developmental roots in Germany and focusses of biopsychosocial support for a wide range of patients, including affective and anxiety disorders, trauma-related syndromes as well as bodily distress in complex medically ill patients. Similarly to patients with severe mental illnesses, this broader group of patients could gain a substantial enhancement of physical health outcomes due to biopsychosocial care e.g. in terms of education and motivation to more adequate health behavior or improvement of their activity level (62).

The treatment structure comes closest to the model of integrated medical/psychiatric care at the Royal Prince Alfred Hospital in Sydney presented by Gertler et al. especially with regard to the small size of the unit (4–5 beds) and indication defined by: “(1) the patient's medical/surgical problem no longer required acute care on the general ward and residual symptoms or continuing physical care would not interfere with the patient's participation in the ward therapeutic program; (2) the patient was sufficiently mobile to attend to his/her personal hygiene; (3) the patient was transferred to the [Medical Psychiatry Unit] on a voluntary basis; (4) the patient was not suffering from drug or alcohol withdrawal, but could have a history of such abuse; […] (6) internists and surgeons who had previously cared for the patient on the general wards would continue to supervise the relevant aspects of the patient's management and be available in an emergency either to consult, or if necessary, accept transfer back to their care.” [(46), p. 27].

Differences exist in the NIPA-team's stronger multidisciplinary approach to specialized professions such as psychologists, art therapists, and social workers in combination with specialized nursing staff. Furthermore, the unique approach of a decentralized structure enables flexibility. Integrated beds across multiple somatic wards ensure specialized biopsychosocial therapy within the somatic clinical setting best suited to patients' physical symptoms. As requested by Caarls et al., this further lowers the barriers to mental treatment and allows for even greater integration and faster availability of care (39). Increased awareness of mental health comorbidities among the internal medical staff is stimulated by proactive case detection, which is ensured by the psychosomatic C-L structures (35). The multimodal treatment of this complex medically ill patient group also brings benefits to the medical and nursing staff in general hospital since their need of support is well-described in the literature [e.g. (32)].

In Germany, there were already models that provided for the psychosomatic care of “interspersed beds” [“Steglitzer Belegbetten-Modell” (63)] or implemented the psychosomatic treatment unit [“Marburg Model” (64)] on somatic wards. The outlined potentials were observed, but also difficulties in maintaining these structures due to a lack of specialized psychosomatic indication options since these models were the stand-alone treatment possibilities back then. The realization of the NIPA concept is only achievable if it is established in addition to a wide range of treatment options offered by highly qualified psychosomatic care, thus closing the gap that has existed to date in the treatment of complexly and chronically ill patients.

Nevertheless, the need for precise definition of admission criteria for integrated units is frequently stated in the literature (39, 48). The occasionally vague nature of these criteria is a risk for confounding communication and connectivity between patients, caregivers, referrers, and payers that we could also observe in the NIPA treatment context. To improve this situation, Caarls et al. formulated five clusters of criteria for the decision making regarding admission to integrated care units, involving patient and organizational variables as well as psychiatric and medical symptoms and treatment capabilities, which can be assessed by a short questionnaire (39). Another promising, systematic approach, the INTERMED method, identifies complex patients and filters out their treatment priorities quickly and economically using a structured interview (65). An improvement of the NIPA concept in this regard is useful and intended. The major economic challenges faced by many Medical Psychiatry Unit approaches (35) are alleviated in the NIPA treatment by, among other things, lower additional costs as the model leverages existing infrastructure.

The results of the treatment show that it was effective in reducing symptoms of depression, anxiety and stress in patients with complex and chronic somatic comorbidity, which support the assumption that NIPA can close a gap in the care of these complex medically ill patients. A considerable proportion of the treatment group showed measurable categorial improvements in symptom burden. The minor changes in somatic symptoms correspond in part to the clinical impression, since the treated group is composed of multiple and often chronically ill patients with often severe somatic comorbidities, thus a significant improvement would not be expected. Furthermore, PHQ 15 measures the distress caused by the occurrence of a wide range of somatic symptoms over a reference period of four weeks. Considering that NIPA treatment has a median duration of about two weeks, the PHQ-15 does not seem to be an appropriate measure to gain meaningful data in terms of assessing change. For this purpose, future evaluations of treatment should involve measurements of health-related quality of life within a shorter reference time period. Due to the naturalistic design, this evaluation study has limitations, especially with regard to the currently still small sample size and a missing control group.

Regarding a more comprehensive evaluation of treatment success, long-term follow-up data are needed to be collected and supplemented by data regarding the quality of life and the frequency of further medical treatment plus a comparison with an appropriate control cohort, which could be obtained, for example, by propensity score matching in the framework of a quasi-experimental design, as presented by Baumgardt et al. (66). Existing evidence indicates the possibility of reducing healthcare utilization and associated socioeconomical costs due to sufficient treatment, e.g. in patients with somatoform disorders (67). Corresponding observations would be of great assistance to many integrated biopsychosocial treatment units such as NIPA. A transfer of this therapy program into other medical disciplines (e.g., surgery) seems to be reasonable. Clinical impressions indicate that the treatment could be helpful for patients of all genders. The greater number of female patients reported for psychosomatic treatments in general was also evident in NIPA treatment. However, due to the small sample, no valid statements can be made on gender aspects.

Naylor et al. state: “From an integrated care perspective, some of the most significant opportunities for innovation lie in building community-facing liaison services that stretch beyond hospital boundaries and work in new ways with community partners” [(36), p. 54]. Especially with regard to many patients' huge dependence on social care or support from the social sector, the strengthening and improvement of collaboration between inpatient and outpatient facilities as well as between the different mental, physical and social caregivers is necessary (48). The NIPA concept, with its focus on low-threshold interventions and the “door-opener” function for further treatment options, e.g., more intensive biopsychosocial treatment in specialized psychosomatic inpatient units or outpatient psychotherapeutic and social psychiatric care, takes important steps in this direction, although these need to be further intensified and extended.

The here presented treatment model requires careful and interdisciplinary coordination, especially with regard to a rapid response to somatic deterioration in the frequent case of multimorbidity. If this challenge is met with effective procedures, one of the great strengths of the concept will have been realized, because this kind of individualized treatment would not be possible in any other context.

## Data Availability Statement

The raw data supporting the conclusions of this article will be made available by the authors, without undue reservation.

## Ethics Statement

Ethical review and approval was not required for the study on human participants in accordance with the local legislation and institutional requirements. The patients/participants provided their written informed consent to participate in this study.

## Author Contributions

PK, EK-K, BS, and CW contributed to the conception and design of NIPA. JF, AD, and MW supported the concept and contributed to the realization in their departments. PK performed the statistical analyses and wrote the first draft of the manuscript. CW wrote sections of the manuscript. All authors contributed to manuscript revision, read, and approved the submitted version.

## Conflict of Interest

The authors declare that the research was conducted in the absence of any commercial or financial relationships that could be construed as a potential conflict of interest.

## Publisher's Note

All claims expressed in this article are solely those of the authors and do not necessarily represent those of their affiliated organizations, or those of the publisher, the editors and the reviewers. Any product that may be evaluated in this article, or claim that may be made by its manufacturer, is not guaranteed or endorsed by the publisher.
